# A new method to standardize CBCT for quantitative evaluation of alveolar ridge preservation in the mandible: a case report and review of the literature

**DOI:** 10.1093/rb/rbv017

**Published:** 2015-10-14

**Authors:** Yang Xia, Lizhe Xie, Yi Zhou, Tianxi Song, Feimin Zhang, Ning Gu

**Affiliations:** ^1^Jiangsu Key Laboratory of Oral Diseases, Nanjing Medical University, Nanjing, 210029, China,; ^2^Jiangsu Key Laboratory for Biomaterials and Devices, Southeast University, Nanjing, 210009, China,; ^3^Beijing Allgens Medical Science & Technology Co., Ltd, Beijing, 100176, China,; ^4^Suzhou Key Laboratory of Biomaterials and Technologies & Collaborative Innovation Center of Suzhou Nano Science and Technology, Suzhou, 215123, China

**Keywords:** CBCT, quantitative evaluation, alveolar ridge preservation

## Abstract

Cone-beam computerized tomography (CBCT) is an effective technique for assessment of changes to the alveolar ridge (AR). However, its accuracy and reliability could be improved by standardization of imaging positions to remain unchanged during measurements. In this study, an alveolar ridge preservation procedure was performed on a left third molar (38) socket by filling it with a radiotransparent synthetic bone graft, mineralized collagen (MC). Photographic, X-ray and CBCT images were captured before and 3, 6 and 12 months after surgery. A new method was developed to standardize CBCT for quantitative evaluation. Obtained CBCT images showed good comparability. The post-extraction alveolar width and height were both over 95% of the original values, but some resorption of the lingual bone wall (>50%) and inter-crestal bone (>30%). It is concluded that an effective positional standardization method was developed for CBCT assessment of AR dimensional changes in the posterior mandible. The use of MC in combination with a collagen membrane improved dimensional preservation of the AR.

## Introduction

Following tooth extraction, the volume of the surrounding alveolar ridge (AR) becomes markedly shrunken as a result of the natural bone remodeling process [1–3]. Preservation of AR dimensions is important because sufficient alveolar bone volume and a favorable architecture are essential to achieving prosthetic reconstructions that are both functionally and aesthetically optimal.

It seems that AR resorption cannot be entirely prevented by alveolar ridge preservation (ARP), but dimensional changes in the AR may be limited by these techniques. Outcomes following ARP treatment were determined by different factors including different anatomical locations [4]. It is reported that, as a result of the surgical removal of impacted third molars, management of the osseous and soft tissues distal to the second molars can be challenging [5]. Guided bone regeneration (GBR) treatments in combination with bone grafts have been used to solve this problem [6]. There have been no reports on the prognosis of post-extraction non-impacted third molars, which are removed in cases such as severe caries or non-functional teeth.

Better bone grafts and more accurate and non-invasive methods for evaluation of ARP are needed. Cone-beam computerized tomography (CBCT) appears to offer an effective, non-invasive and relatively low radiation technique for assessment of dimensional changes in the AR [7]. However, standardization is necessary for this technique to be truly reliable; images should always be captured and reconstructed at identical positions [8] in order to get identical CBCT sections for reliable quantitative measurements. Moreover, it is well known from studies in medical and dental radiology that various observers may arrive at different results when examining the same radiographs.

Anatomic landmarks are good choice to determine a fixed position on both real human body and images [9]. They are used to correct the linear length before CBCT images are undergone measurements. Lascala et al. [10] performed linear measurements between anatomic landmarks in dry human skulls scanned with the NewTom 9000 device. So it is believed that anatomic landmarks on CBCT images could be useful to obtain the same sections of the same patient if each CBCT was performed at exactly the same position.

Changes in the AR contour observed on the CT image should be interpreted with caution if the graft material possesses radiopaque characteristics (e.g., DBBM) [11]. Mineralized collagen (MC) is a synthetic bone graft with an intricate hierarchical structure of mineralized collagen fibers formed by nano-sized hydroxyapatite (HA) crystals. During self-assembly, the mineralized collagen nano-fibrils align parallel to each other to form mineralized collagen fibers [12]. The hierarchical structure of MC has been shown to resemble natural bone structure [13]. It is radiotransparent, thus will not disturb the characterization of new bone regeneration after transplantation. Moreover, it has been used as a bone graft substitute for anterior cervical intersomatic fusion [14], and was reported to be good in osteogensis [15]. Here we applied it in dental field.

Clinical data on the standardization of radiographic measurements is lacking. In the current study, a method using anatomic landmarks on CBCT images to standardize the measurements of CBCT sections was developed, and then evaluated in the case of an extracted human non-impacted third molar whose socket was grafted with MC and covered by a resorbable collagen membrane. The ARP effect of the graft was compared with natural healing (NH).

## Case report

The study protocol was approved by the Ethics Committee of Institute of Stomatology, Nan Jing Medical University. The patients were treated in accordance with the Helsinki declaration. Informed consent of the patient was obtained after explaining the clinical procedures, risks involved and benefits and clarifying all questions raised by the patients.

Tooth 38 of a 30-year-old female was extracted. The socket was filled with MC (Allgens Co., Ltd., Beijing, China) and then covered with a collagen membrane (Bio-gide, Geistlich, Switzerland). As a control, tooth 38 of a 24-year-old female, which was in almost the same position, was extracted and left untreated to heal naturally. Both the teeth were decayed, non-functional and in good contact with the adjacent tooth 37 (Figs 1 and 7a).

**Figure 1 a and b.**
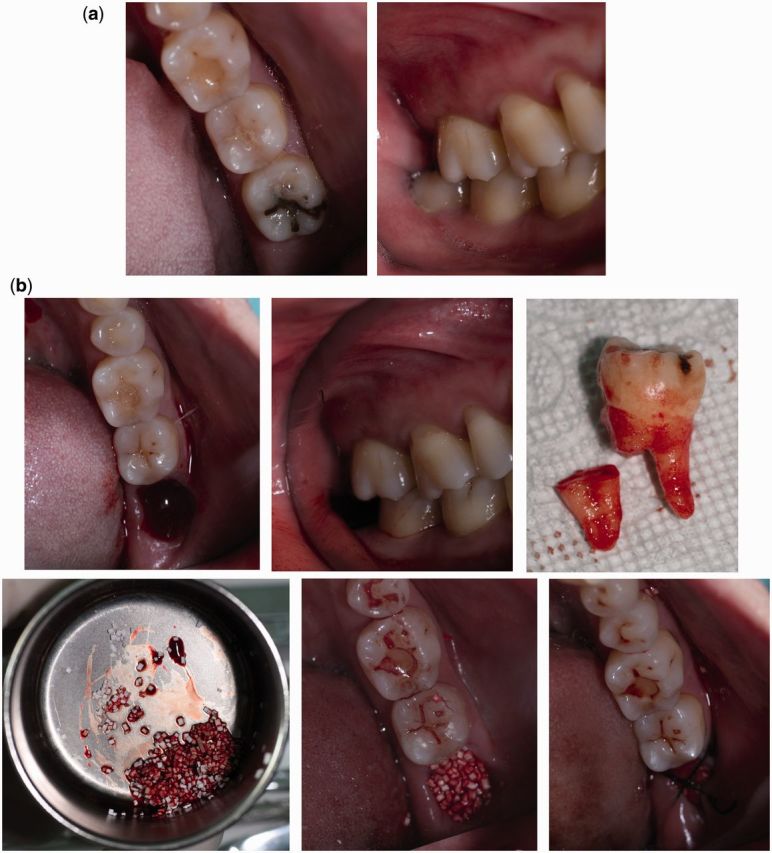
Clinical photographs illustrating the condition of experimental site: (**a**) before tooth extraction; (**b**) during tooth extraction.

**Figure 1c.**
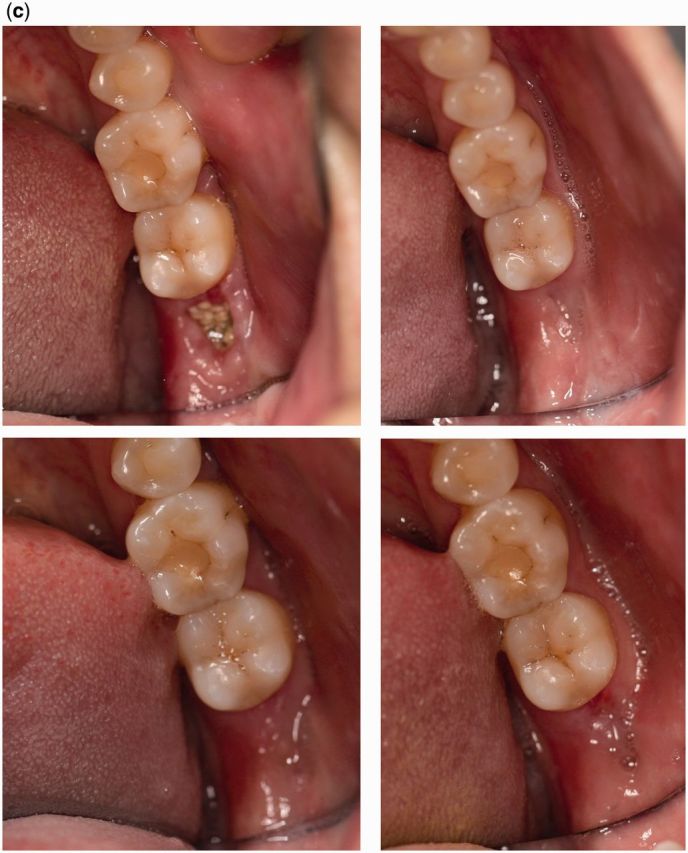
Clinical photographs illustrating the condition of experimental site: (c) after tooth extraction. Top left: 2 weeks after extraction; Top right: 3 months after extraction; Below left: 6 months after extraction; Below right: 12 months after extraction.

Atraumatic extractions were performed under local anesthesia by a clinician with 10 years’ experience in the specialty. MC pre-wetted with the patient’s blood was used to completely fill the socket from the crest of the alveolus to the apex. A collagen membrane, extending 3–4 mm beyond the AR border, was used to cover the experimental region. The surgical procedure was completed with a cross-mattress suture. Care was taken to ensure perfect retention of the scaffold and membrane. Part of the membrane was exposed since primary closure was not performed. The same procedure was undertaken on the control site without using grafts or membranes.

Intraoral photographs at different time points are shown in Fig. 1. There was no obvious sign of inflammation. Complete epithelialization of the socket was observed after 3 months, and thereafter, the defect remained fully covered by gingiva. No obvious macroscopic changes to the gingiva were found post-surgery. The oral hygiene of the patient was good.

**Figure 2.**
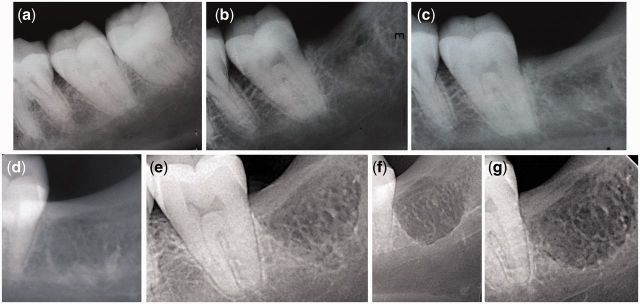
X-ray images of experimental site at different time points. (a): before extraction; (b): immediately after extraction; (c): after suturing; (d): 2 weeks after extraction; (e): 3 months after extraction; (f): 6 months after extraction; (g): 12 months after extraction.

X-ray images at different times are shown in Fig. 2. The margin of the bone defect remained clearly discernible 2 weeks post-extraction, but after 3 months, it became diffuse and faint. These changes in X-ray densities, presenting as unclear socket outlines fading into the peripheral area, indicated trabecular hyperplasia and the activity of osteoblasts and osteoclasts. However, the densities remained low after 12 months, indicating that the regenerated tissues were not as mineralized as mature bone.

For CBCT, the midline laser beam of the CBCT system was adjusted to the midsagittal plane of the skull. The horizontal laser beam was parallel to the Frankfort plane of the skull. The scan time was 18 s and the voxel size was 0.3 mm (5.56 mAs, 110 kVp). The raw data set was reconstructed using the CBCT software (QRNNT 2.17, Quantitative Radiology, Verona, Italy).

**Figure 3.**
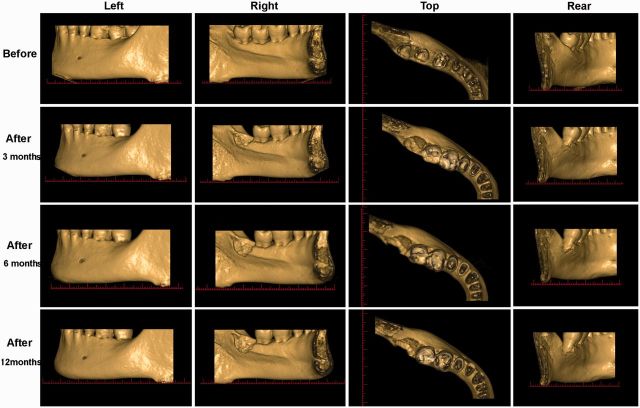
Three-dimensional CBCT reconstructions of experimental site from left, right, top and rear perspectives at different time points.

Reconstructions of left mandible (Fig. 3) from the left perspective, where no obvious change was observed, were used as references for standardization. From the right perspective, the lingual socket wall was above the mylohyoid line of the mandible before extraction. The tooth was shown to be close to the lingual side. It gradually reduced to (3 months) and then below (6 and 12 months) the mylohyoid line after extraction. Resorption of the lingual bone wall can also be observed from the top perspective. From the rear perspective, continuous bone loss distal to tooth 37 could be seen.

**Figure 4.**
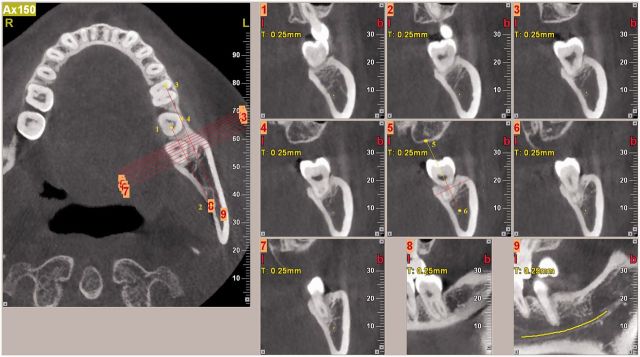
CBCT sections for quantitative analysis (experimental site before extraction). Typical lines on chosen sections to determine the position were shown. The reference line is between point 5 and 6. Lines for alveolar ridge width and height are marked on the reference line.

The CBCT sections used for quantitative analysis are shown in Fig. 4. The coronal plane was set parallel to the lower side of the mandible at the section where the images of point 1 (distal pulp cavity of tooth 37) and point 2 (mandibular foramen) were clearest. The sagittal plane was set over the line between the above two points, and the section was marked as image 8. From mesial to distal, 60 axial images were obtained when slice thickness was set at 0.25 mm. Slices 11, 13, 15, 17, 19, 22 and 25 were chosen, and marked as image 1, 2, 3, 4, 5, 6 and 7. Another sagittal plane marked as image 9 was made over the line between point 3 (the mesio pulp horn of tooth 36) and point 4 (the buccal side of tooth 37), on which the mandibular nerve was marked.

**Figure 5.**
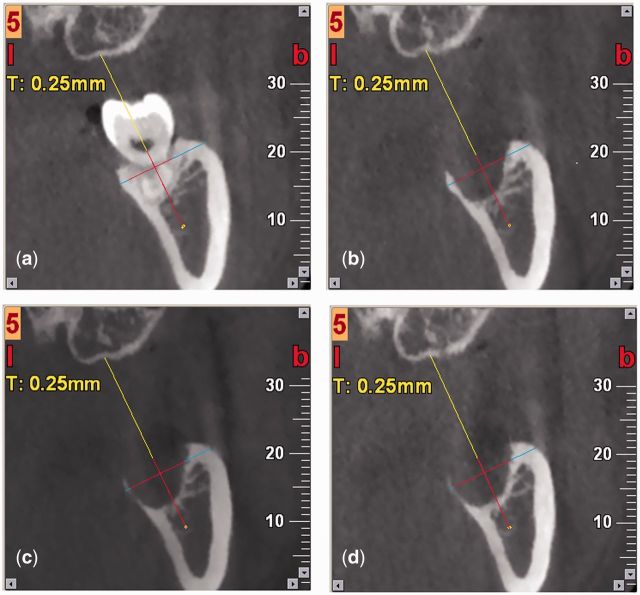
CBCT of experimental site for quantitative analysis of alveolar dimensional changes in horizontal and vertical directions. (a): before extraction; (b): 3 months after extraction; (c): 6 months after extraction; (d): 12 months after extraction. Reference line, lines for alveolar height and width, and lines for buccal and lingual bone wall thickness are marked separately.

On image 5, a reference line was set between point 5 (a notch on the maxilla) and point 6 (mandibular nerve) (Fig. 4). Alveolar width was measured by its horizontal projection on the reference line. Alveolar height was represented by vertical projection from the top of the buccal (lingual) bone wall to the mandibular nerve on the reference line. The thickness of buccal and lingual bone wall was measured on the lines representing alveolar width (Fig. 5).

**Figure 6.**
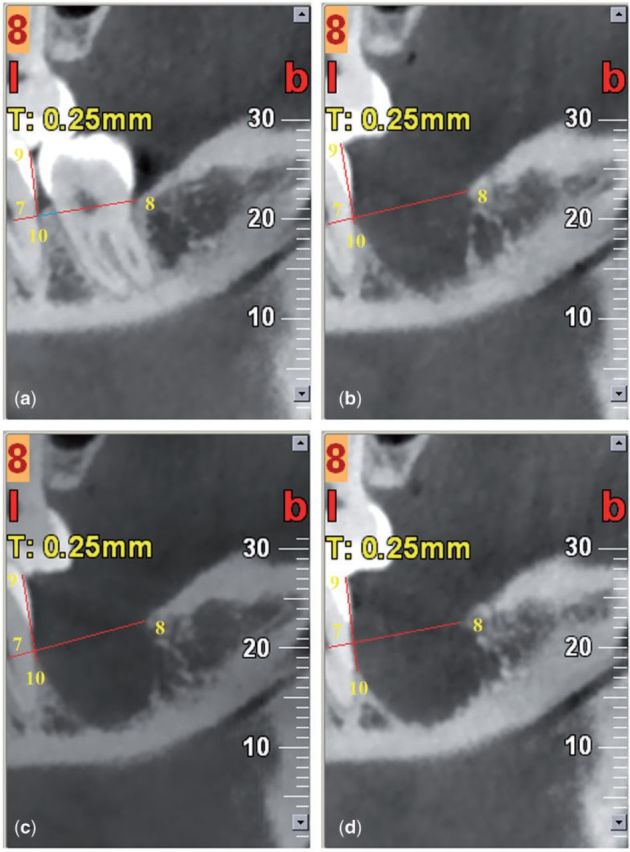
CBCT of experimental site for quantitative analysis of dimensional changes of the alveolar ridge in the mesio-distal direction and in the inter-crestal bone distal to tooth 37. (a): before extraction; (b): 3 months after extraction; (c): 6 months after extraction; (d): 12 months after extraction.

On image 8, mesio-distal bone changes were measured by the distance between point 7 (the distal pulp floor of tooth 37) and point 8 (the distal socket edge) (Fig. 6). Height changes of inter-crestal bone distal to tooth 37 were recorded; they were represented by the distances between point 9 (distal occlusal contact points of upper and lower tooth 37) and point 10 (the crestal bone of tooth 37 distal roots).

**Figure 7.**
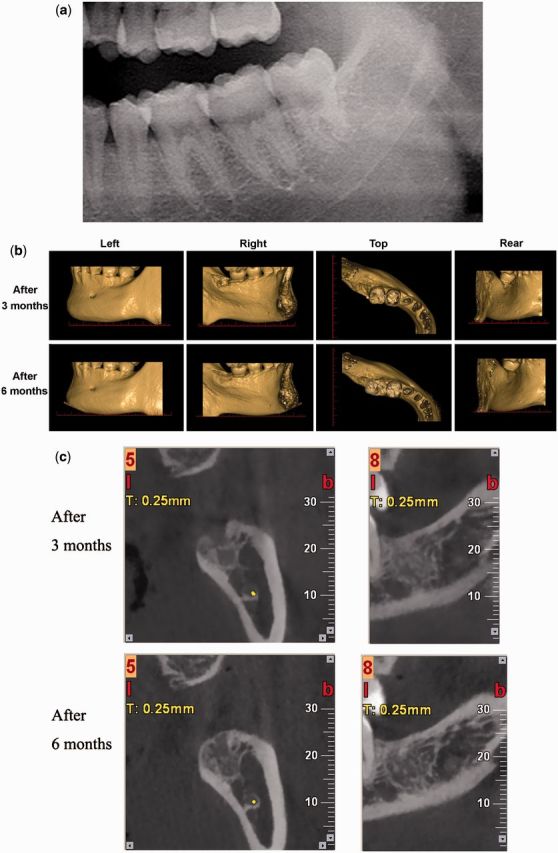
Radiographs of control site. (a) X-ray image before extraction; (b) CBCT 3D reconstructions at 3 and 6 months after extraction; (c) CBCT sections 3 months and 6 months after extraction.

Radiographic measurements were taken by three trained observers, with no knowledge of the actual physical dimensions. The results were represented as the mean value of five individual measurements, which were normalized to the length of the reference line. Images were analyzed using Image-Pro Plus 6.0. Changes in dimensions (CID) were expressed as the normalized percentage of the pre-extraction value as follows:

$$\begin{array}{l} \text{Normalized ratio}=\frac{\text{length of reference line after}}{\text{length of reference line before}}\\ \text{CID}\%=\frac{\frac{\text{alveolar dimensions after}}{\text{normlized ratio}}}{\text{alveolar dimensions before}}\times 100\% \end{array}$$

The CID of alveolar ridge is shown in Table 1. The normalized ratios were all close to 1, showing good consistency. The remaining percentage for both AR width and height was more than 95%, indicating excellent efficacy in maintaining AR dimensions. AR width seemed to be better preserved than height, whereas in natural healing the shrinkage of width is more obvious [16]. The changes in bone wall thickness were much greater; resorption of buccal bone wall was nearly 20%, while that of lingual bone wall was over 50% at 6 months post-extraction. Both vertical and horizontal resorption of alveolar bone distal to tooth 37 was detected.

**Table 1.** Changes in dimensions (CID) of alveolar ridge by CBCT measurements at different time points

	Before	3 months after	6 months after	12 months after
Reference line (mm)	27.96	27.96	27.63	27.98
Normalized ratio	**–**	1	0.988	1.0007
Width (mm)	13.81	13.76	13.59	13.76
CID (%)	**–**	99.64	99.64	99.57
Height (mm)	11.95	11.66	11.49	11.56
CID (%)	**–**	97.57	97.32	96.65
Buccal wall (mm)	4.81	3.96	3.84	3.84
CID (%)	**–**	82.32	80.67	79.83
Lingual wall(mm)	1.25	1.13	0.57	0.57
CID (%)	**–**	90.40	46.40	45.60
Mesio-distal (mm)	12.70	14.16	13.91	13.26
CID (%)	**–**	111.50	110.24	104.33
Inter-crestal bone (mm)	6.64	7.95	8.72	9.30
CID (%)	**–**	119.73	132.98	139.90

The data collected from the control patient is presented in Fig. 7. Identical 3D and 2D CBCT images were obtained and analyzed using the same standardized method. The result of the experimental site was compared with that of the control site to find the differences. Compared Fig. 3 with Fig. 7b, it was obvious that the control site shrank in width, but no distinct bone loss distal to tooth 37. It was consistent with the comparison in 2D sections (Figs 5 and 6 with Fig. 7c), which also showed notable shrinkage in ARP width. The reduction in height was not clear by naked eye. Generally, AR resorption was substantial in the control site and it was greater horizontally than vertically. Better bone regeneration could be seen in the control site as indicated by increased radiopacity.

## Discussion

The aim of the present study was to develop a method of standardizing the dimensional measurements used for CBCT evaluation of AR preservation in human. CBCT provides 3D and cross-sectional views of the jaws and linear measurements of the resulting images are used in presurgical implant planning for determination of alveolar height and width, and consequently, the required implant size. Linear measurements are also used in orthodontic analysis and the determination of jaw-tumor size. Studies have shown that 94% of linear CBCT measurements are accurate within 1 mm [17–19]. So, it should be the best method for measurement of AR volume and morphology in a non-invasive and accurate manner.

In this study, the images were always captured and reconstructed at identical position for the purpose of standardization using the reference plane determined by anatomic landmarks on CBCT sections. The accuracy of measurement distances in patients can be affected by a reduction in image quality due to soft tissue attenuation, metallic restorations and patient movement. To increase accuracy and repeatability, clear and relatively fixed points representing the relevant anatomic structures were chosen and used to make lines and sections for corrections and measurements. This method was effective for obtaining identically positioned images as shown in Figs 4 and 7. These accurately matched images were critical for the subsequent quantitative measurements.

Lambert et al. [20] also developed an approach to assessing alveolar ARP procedures in humans by CBCT, and applied to a series of cases in the upper anterior maxilla. CT scans in their studies were standardized by a custom-made template. Comparatively, the method developed in this study was easier to carry out because we did not need any specially made devices. The chosen points for calculation were fixed anatomic points in the posterior area. These points were unique and easy to determine on CBCT images. And they were constant among normal people. Nine sections obtained by lines 1–2, 3–4 and their seven vertical lines were identical at each time points. Reference line 5–6 was used for correction before measurements to ensure the consistency among different images. And the result (close to 1) proved it. Moreover, the measurements can be done from three dimensions. So it was assumed that our method was accurate, repeatable and effective. And it could be used for CBCT assessment of AR dimensional changes in the posterior mandible.

The inferior alveolar nerve was chosen as one of the constant points (point 6) from which to determine the reference line for normalizing measurements; as it is one of the determinants of the bone height available for dental implants, and the objective was to develop a method which can be extended to sites intended for an implant. The final results were reported as percentage. It could be more useful for clinicians to report tendencies of changes in alveolar dimensions rather than exact values.

Several methods for ARP have been investigated [21–23], including socket grafting with autogenous bone [2], demineralized freeze-dried bone allografts (DFDBAs), xenografts (e.g., deproteinized bovine-bone mineral, DBBM) [24], alloplasts [25] and bone morphogenic proteins (BMPs) [26]. Guided bone regeneration (GBR) with or without bone grafts has also been evaluated [27–29]. The buccal bone plate is almost entirely comprised of bundle bone, and tends to exhibit marked osteoclastic resorption in the coronal region of the socket [30, 31]. This agrees with what was observed in our naturally healed control whose AR width was decreased. The resorption of buccal bone wall proceeds with a marked reduction in the horizontal dimension (predominantly) [32]. But in the experimental site, the resorption percentage of lingual bone wall was greater than that of buccal side. Possible reasons may attribute to the difference in their original bone wall thickness. The experimental tooth was close to the lingual side. So its buccal bone wall, which was near mandibular external oblique ridge, was much thicker than lingual bone wall. The AR shape of experimental site remained almost unchanged. Thus, when the bone wall underwent the same shrinkage in thickness, lingual side presented more obviously.

The occurrence and manner of socket healing in the control, which was initiated from the apical and lateral regions of the extraction socket walls, was in agreement with previous studies in which soft tissue closure was not performed [16, 33]. The poor bone regeneration at the ARP site may have been due to the early exposure of the membrane which could lead to infection and disintegration, followed by loss of bone in the grafted area [34]. And it is reported that patient over 25 years of age to be more likely to develop an intrabony defect after extraction of an impacted third molar without preventive procedures [35]. In the current study, the experimental patient was older than 25, while the control patient was younger than 25. This difference should be noticed and excluded in further study.

The quality of new socket tissue varies widely, based on the type of bone graft used. Remnants of the grafts often interfered with the normal healing process in line with preclinical results [16, 36]. At the histological level, evidence relating to the benefits of ARP is conflicted and ARP does not appear to promote hard tissue regeneration routinely [27, 37]. In addition, some graft materials may interfere with healing [21, 38]. This is the first report of the application of MC in human alveolar bone. The bone density after 12 months seemed not as good as that in the untreated socket; presenting as low densities in X-ray and CT images. As mentioned above, one possible reason may be the exposure and early resorption of the collagen membrane. It can lead to the early loss of the transplanted bone grafts, thus resulted in the poor bone regeneration and even inter-crestal bone resorption (Fig. 6). Better coverage of the extraction by the membrane or the gingiva, and the cubic block shaped MC would be the solutions to achieve better ARP results. Another possible reason may be the difference in patient’s age. Younger patient showed better bone regeneration results. However, there were almost no dimensional changes in the ARP after extraction in contrast to the control group.

It is doubtful whether the ARP technique should be considered successful when it only preserves the external contour of the AR, with the newly formed tissue being of an inferior quality and quantity to that which is naturally achieved following tooth extraction. The small sample sizes and absence of histological analysis (for the benefit of the patient) made it difficult to provide a reliable evaluation of the ARP procedure and the effects of the grafted materials. Further studies using a larger sample group will be carried out to elucidate the determinant factors in ARP among the patients’ age, anatomic conformation, and the properties of bone grafts. And sites where would be implanted with dental implants will be tested, so the quality of the new bone can be examined histologically and compared to natural healing. The results can reveal the true effects of MC in ARP.

## Conclusion

A non-invasive method to evaluate alveolar dimensional changes by positionally standardized measurements of CBCT cross-sections was shown to be highly accurate, repeatable and scalable. While keeping in mind the limited number of patients studied, we conclude that standardized analysis of CBCT images is an accurate way to assess dimensional changes in the AR. The grafting of mineralized collagen covered by a collagen membrane into a socket immediately after tooth extraction allowed for almost complete preservation of alveolar dimensions.

## Funding

This work was supported by grants from the Outstanding Medical Academic Leader Program and Creative Team of Jiangsu Province, the Project Funded by the Priority Academic Program Development of Jiangsu Higher Education Institutions (PAPD, 2014-37), the National Natural Science Foundation of China (No. 81400486), the Natural Science Foundation of Jiangsu Province (No. BK20140911), the Postdoctoral Science Foundation of Jiangsu Province (No. 1402044B), the China Postdoctoral Science Foundation (No. 2015M571647). 

*Conflict of interest statement*. None declared.
